# Supplemental Blue Light Frequencies Improve Ripening and Nutritional Qualities of Tomato Fruits

**DOI:** 10.3389/fpls.2022.888976

**Published:** 2022-06-09

**Authors:** Rui He, Jingjing Wei, Jiye Zhang, Xin Tan, Yamin Li, Meifang Gao, Houcheng Liu

**Affiliations:** College of Horticulture, South China Agricultural University, Guangzhou, China

**Keywords:** blue light, supplemental frequencies, ethylene release, lycopene, ripening

## Abstract

Tomatoes (*Solanum lycopersicum* L. Micro-Tom) were grown in a plastic greenhouse. When plants anthesis, the 100 μmol m^–2^ s^–1^ blue light-emitting diode (LED) light (430 ± 10 nm) was supplemented from 6:00 to 18:00. There were 5 treatments, which contained different blue light frequencies with the same intensity: S6 (30 min blue light and 30 min pause), S8 (30 min blue light and 15 min pause), S10 (30 min blue and 8 min pause), S12 (continuous blue light for 12 h), and control (CK) (natural light, without any supplemental light). Agronomic traits and nutritional qualities of tomato fruits were measured at 30, 34, 38, 42, and 46 days after anthesis (DAA), respectively. Different frequencies of supplemental blue light could accelerate flowering of tomato plants and promote fruit ripening about 3–4 days early *via* promoting ethylene evolution of fruits, which significantly facilitated the processes of color change and maturity in tomato fruits. The contents of lycopene, total phenolic compounds, total flavonoids, vitamin C, and soluble sugar, as well as the overall antioxidant activity of tomato fruits were significantly enhanced by all the supplemental blue light treatments. In all, different frequencies of supplemental blue light prominently reinforced the antioxidant levels and nutritional qualities of tomato fruits, especially lycopene content, and S10 was more optimal for tomato fruits production in a plastic greenhouse.

## Introduction

Tomato (*Solanum lycopersicum* L.), one of the most favorite fruit vegetables consumed popularly worldwide, is considered as superb sources of dietary antioxidants due to its rich carotenoids content (Dorais et al., 2008). Among all the sorts of carotenoids, lycopene is one of the most extraordinary antioxidant properties in tomato, which is directly responsible for tomato color (Górecka et al., 2020).

Light is important for plants due to its vital roles in morphology, physiology, and development from seed germination to flowering (Kami et al., 2010). Light could trigger various cellular responses in plants when it was perceived and processed by complicated photoreceptors, leading to physiological and metabolomic alterations of plants (Galvão and Fankhauser, 2015). Supplemental lighting is critical to meet the demands for high irradiance during plant growth and fruit development in greenhouse, particularly in the weak light environment (Fanwoua et al., 2019). Light-emitting diodes (LEDs) light has a series of advantages such as narrow bandwidth, long lifetime, low heat output, low weight, high energy efficiency, and low electricity consumption compared to traditional light sources. Therefore, LEDs as environmental and effective means were used in greenhouses for commercial production of horticultural crops (Lazzarin et al., 2020).

It is noteworthy that blue LED light enhances secondary metabolites in plants, including ascorbate, total phenolic, anthocyanin, flavonoid contents, and antioxidant activity (Huché-Thélier et al., 2016; Qian et al., 2016; Jung et al., 2018, 2021). Blue light is more efficiently absorbed by photosynthetic pigments than other spectral regions. Supplemental blue light promoted early tomato flowering and fruit color change and had a significant effect on the accumulation of lycopene (Xie et al., 2019). Numerous studies have focused on the impacts of different light intensities or qualities on growth and development of plants in closed controlled environments (Ji et al., 2020; Kim et al., 2020; He et al., 2021). However, the effects of intermittent light on plant growth are still limited. Study showed that the seedlings of Red Russian Kale, Purple Top Turnip, and Ruby Queen Beet cultured under 5 s on/off pulses were roughly comparable developmentally to seedlings grown under 12 h on/off pulses (Song et al., 2019). Meanwhile, higher shoot biomass of lettuce was brought by the intermittent red blue (RB) irradiation of L/D (4), L/D (6), and L/D (8) compared with the continuous RB light (Chen and Yang, 2018). Moreover, higher quantum efficiency of photosystem II and crop productivity were observed in tomato plants under pulsed light than continuous light (Olvera-González et al., 2013). Intermittent irradiation displayed higher biomass and better quality than continuous irradiation that might be related to dark-induced positive regulations, since higher activities and gene expressions of some enzymes were upregulated under the darkness (Klopotek et al., 2016). Thus, the application of pulsed light to light periods might provide a new perspective for regulating plant growth and development and might be beneficial for energy saving by supporting comparable growth with significantly lower daily light integrals.

In this study, the effects of blue light supplementation at different frequencies on tomato (Micro-Tom) fruit ripening and nutritional quality were explored, in order to provide a theoretical basis for efficient light supplementation and high-quality tomato production in protected cultivation.

## Materials and Methods

### Plant Materials and Light Treatments

The experiment was performed in a plastic greenhouse at the College of Horticulture of South China Agriculture University. Tomato (*Solanum lycopersicum* L. Micro-Tom) seedlings were cultivated on sponge blocks (2 cm × 2 cm × 2 cm) with a Yamasaki tomato nutrient solution, which was refreshed every week. The seedlings with three fully expanded true leaves were transplanted into a customized hydroponic system at a density of 20 plants per plate (80 cm × 55 cm × 11 cm), which were considered as one replicate. All the treatments included three replicates. In the plastic greenhouse, photosynthetic photon flux density (PPFD) at 12:00 a.m. was 500 ± 50 μmol m^–2^ s^–1^, ambient temperature was 25 ± 5°C, and air humidity was 35–90%.

Tomato seedlings grown under natural light were used as control (CK) (without any supplemental light). There were four frequencies of supplemental blue light treatments: S6 (30 min blue light and 30 min pause), S8 (30 min blue light and 15 min pause), S10 (30 min blue light and 8 min pause), and S12 (continuous blue light for 12 h). Supplemental lighting was blue LEDs (430 ± 10 nm, Chenghui Equipment Corporation Ltd., Guangzhou, China). The supplementary light intensity was set uniformly at 100 μmol m^–2^ s^–1^ PPFD for 12 h per day (6:00–18:00) at the onset of anthesis. The total number of flowerings on each treatment (60 plants) was recorded everyday. Fruits were harvested at five developmental stages (thereafter referred to as DAA30, DAA34, DAA38, DAA42, and DAA46), at 30 (mature green), 34 (turning), 38 (orange), 42 (orange), and 46 (red ripe) days after anthesis (DAA), respectively. All the fruits were harvested at the same time of the day with three biological replicates (each replicate consisted of five fruits from different plants). Seeds, jelly, and placenta were removed for each fruit and the pericarp was sampled and immediately frozen in liquid nitrogen and then stored at −80°C.

### Fruit Color Measurement

The changes of fruit color were determined by the Konica Minolta CM-2003d colorimeter. The L*, a*, and b* spaces were processed to obtain the hue angle value. The hue angle was calculated according to the following equations: hue = tan^–1^ (b*/a*), the chroma value, which was determined by [(a*)^2^ + (b*)^2^]^1/2^, represents (Ecarnot et al., 2013).

### Ethylene Release Measurement

The tomato fruits were placed in airtight vials and equilibrated at 25°C for 30 min, with four biological replicates for each treatment (each replicate composed of five fruits from different plants). Gas samples were collected and analyzed *via* gas chromatography (Unicam Analytical, Cambridge, United Kingdom) equipped with an activated alumina column (150 mm × 6 mm) and controlled by Unicam 4880 chromatographic data processing software (Unicam Analytical, Cambridge, United Kingdom). The temperature was as follows: column, 110°C; ejector, 108°C; and detector, 160°C. Ethylene was identified *via* co-migration with an ethylene standard and quantified with reference to a standard curve for ethylene concentration.

### Determination of Antioxidant Capacity and Nutritional Quality

Lycopene extraction and quantification were carried out as described in a study by Xie et al. (2019) with some modifications. Samples were weighed (0.5 g fresh weight) and then immersed in 1.5 ml of a mixture of methanol/tetrahydrofuran (1:1, v/v). The supernatant was collected after centrifugation (1,200 *g*, 10 min, 4°C). Quartic supernatant was collected and then evaporated to dryness under nitrogen gas stream. Then, 1.5 ml methyl tert-butyl ether and methanol solution mixture (V:V = 500:475) were added to the concentrated extract in the vials. The final extract was resuspended in 1.5 ml of 500:475 methyl tert-butyl ether: methanol, filtered through 0.22 μm membrane filter, and analyzed *via* high-performance liquid chromatography (HPLC) (Waters Corporation, Milford, MA, United States) equipped with a model 2489 UV/Vis detector. Samples (20 μl) were separated at 30°C on a reversed-phase C30 column (5 μm, 250 mm × 4.6 mm, Waters Corporation, Milford, MA, United States). The mobile phase consisted of methanol (A) and methyl tert-butyl ether (B) and flow rate was 3.0 mol⋅l^–1^. Chromatograms were recorded at 450 nm. The lycopene was identified by its retention time and spectral data as compared by standards.

Polyphenol content was measured according to the Folin–Ciocalteu assay (Singleton et al., 1999). Fresh samples (0.5 g) were extracted with 8 ml ethanol. After centrifugation for 10 min at 3,000 *g*, 1 ml supernatant was mixed with 0.5 ml folin-phenol, 11.5 ml 26.7% sodium carbonate, and 7 ml distilled water. After 2 h, the absorbance was read at 760 nm by UV-spectrophotometer (Shimadzu UV-16A, Shimadzu, Japan).

Total flavonoid content was determined following to a study by Li et al. (2011). 1 ml extract solution (the extraction for flavonoid content determination was similar to that of the polyphenol content determination) was mixed with 0.7 ml 5% NaNO_2_. Standing for 5 min, the reaction solution was added to 0.7 ml 10% Al(NO_3_)_3_ and 5 ml 5% NaOH and 10 min later, the absorbance was read at 510 nm by UV-spectrophotometer.

The 2,2-diphenyl-1-picrylhydrazyl (DPPH) radical scavenging rate was measured according to a study by Tadolini et al. (2000). 2 ml supernatant (the extraction for flavonoid content determination was similar to that of polyphenol determination) was mixed with 2.0 ml DPPH solution (0.0080 g DPPH in 100 ml ethanol). The absorbance was read at 517 nm by UV-spectrophotometer.

The value of ferric-reducing antioxidant power (FRAP) was determined following to a study by Benzie and Strain (1996). 0.4 ml supernatant (the extraction for FRAP determination was similar to that of polyphenol determination) was mixed with 3.6 ml mixed solution (0.3 mol⋅l^–1^ acetate flavon; 10 mmol⋅l^–1^ TPTZ; 20 mmol⋅L^–1^ FeCl_3_, 10:1:1, v:v:v) and incubated at 37°C for 10 min. The absorbance was read at 593 nm by UV-spectrophotometer.

Soluble sugar content was determined using the sulfuric acid anthrone method (Kohyama and Nishinari, 1991). Fresh sample (0.5 g) was mixed with 8 ml 80% ethanol and then stand on a water bath (80°C) for 40 min. Then, 0.2 ml supernatant was collected and mixed with 0.8 ml distilled water and 5 ml sulfuric acid anthrone. The mixed solution was boiled for 10 min. The absorbance was measured at 625 nm by UV-spectrophotometer.

Soluble protein content was determined using the method of Coomassie Brilliant Blue G-250 dye (Blakesley and Boezi, 1977). Fresh sample powder (0.5 g) was mixed with 8 ml distilled water and then centrifuged at 3,000 *g* for 10 min at 4°C. After that, 0.2 ml supernatant was mixed with 0.8 ml distilled water and 5 ml Coomassie Brilliant Blue G-250 solution (0.1 g⋅l^–1^). Then, 5 min later, the absorbance was read at 595 nm by a UV-spectrophotometer.

The vitamin C content was determined using the method of Chanwitheesuk et al. (2005) with some modifications. Fresh samples (0.5 g) were homogenized with 25 ml oxalic acid ethylenediaminetetraacetic acid (EDTA) solution (0.2 mol⋅l^–1^ EDTA and 0.05 mol⋅l^–1^ oxalic acid). Next, 10 ml extracting solution was added to 1 ml 3% HPO_3_, 2 ml 5% H_2_SO_4_, and 4 ml 5% H_8_MoN_2_O_4_. After 15 min, the absorbance was measured at 705 nm by UV-spectrophotometer.

### Statistical Analysis

All the values are shown as the means of three replicates with SD. Statistical analysis was performed by the Duncan’s test at *p* < 0.05 using SPSS version 25.0 software (SPSS Incorporation, Chicago, IL, United States). The figures were prepared by Origin Professional software version 2019b (OriginLab, Northampton, MA, United States).

## Results

### Flowering Response to Various Supplemental Blue Light Frequencies

**FIGURE 1:**
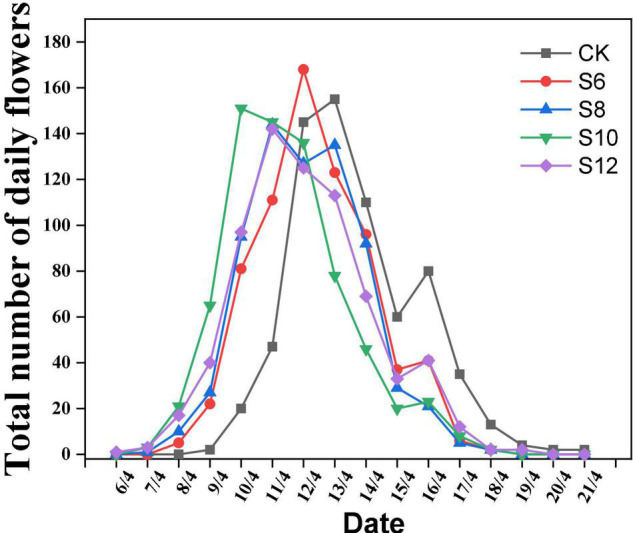
Effect of different supplemental blue light frequencies on flowering of tomato. The data representing the total number of daily flowers in all the plants (*n* = 60) for each treatment.

All the supplemental blue light treatments were positively correlated with earlier flowering in tomatoes, whereas the days to anthesis did not significantly differ among different blue light frequencies treatments. The time of the first flower appearance of tomato plants and daily blooming numbers were recorded (Figure 1). It was found that 1, 2, 2, and 3 days flowering earlier under S6 (8/4/), S8 (7/4), S10 (7/4), and S12 (6/4), respectively, compared to CK (9/4). As for daily blooming number of tomato plants, S10 treatment was earlier (9/4) to reach the peak (flowering number > 60), followed by S6, S8, and S12 treatment. Therefore, these indicated that supplemental blue light was benefit to promoting flowering in tomato plants.

### Supplemental Blue Light Frequencies Treatments Lead to Early Fruit Ripening

**FIGURE 2:**
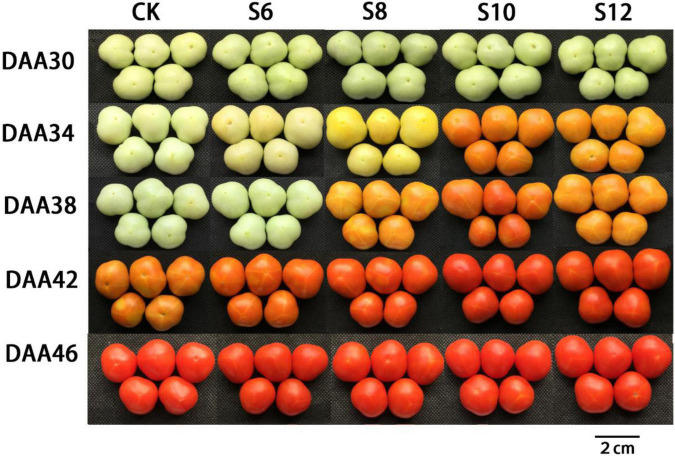
Changes in fruit characteristics during tomato ripening under different stage and different supplemental blue light frequencies.

To investigate whether blue light supplementation impacted tomato fruit ripening initiation and maturity, the days from anthesis to mature stage were counted and the ripening-associated changes in fruit color were monitored (Figure 2). Fruits in CK required about 42 days on average to turn yellow, while fruits under S6, S8, S10, and S12 ripened at 42, 38, 34, and 34 days, respectively, about 0–8 days earlier than CK (Figure 2). The results indicated that the ripening-associated fruit color transition occurred earlier in blue light-treated fruits than in CK fruits, particularly under S10 and S12.

**FIGURE 3:**
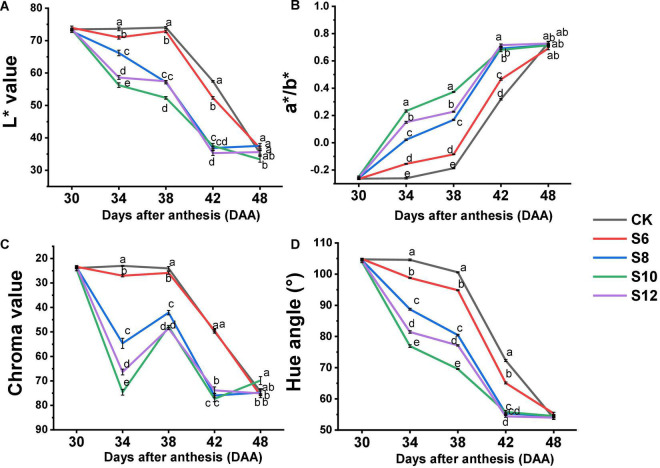
Effect of different supplemental blue light frequencies on surface color values [L*, a*/b*, chroma, and hue angle; **(A–D)**] of tomato fruit during different growth stages. Values are means ± SD (*n* = 3). Different letters denote significant differences among treatments at each sampling day after anthesis separately (*p* ≤ 0.05).

Color change from green to red is a very important indicator of tomato fruit ripening, which is well revealed by L* (lightness), a*/b*, chroma, and hue angle values (Figure 3). The L* value among the treatments displayed no obvious difference at DAA30, while continuously decreased in all the fruits from DAA38 to DAA42 and the highest and the lowest of L* values were found in CK and S10, respectively (Figure 3A). The a*/b* value subsequently increased with tomato fruit ripening, fruits under the blue light treatment exhibited the deeper color and yellowness from DAA34 to DAA42, and the lightest color and the highest yellowness were observed in the S10 (Figure 3B). The chroma value in all the treatments consistently decreased, with the CK maintaining the highest value and the S10 kept the lowest value from DAA34 to DAA42 (Figure 3C). The hue angles of fruit consistently decreased from 100 to 50° with fruit ripening (Figure 3D) and the most drastic drop was observed in S10, which displayed the descending rate as follows: S10 > S12 > S8 > S6 > CK.

**FIGURE 4:**
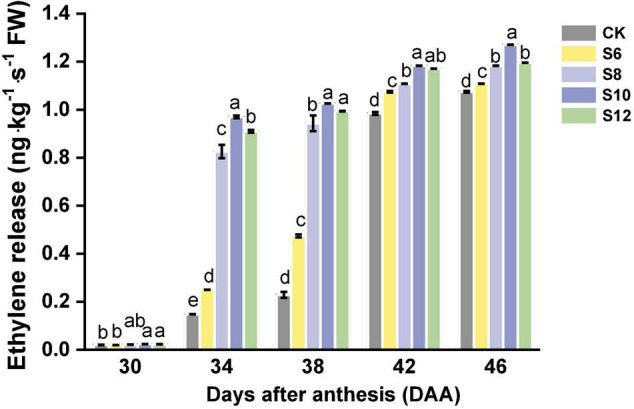
The ethylene release during tomato fruit ripening under different supplemental blue light frequencies. Values are means ± SD (*n* = 3). Different letters denote significant differences among treatments at each sampling day after anthesis separately (*p* ≤ 0.05).

Tomato is climacteric fruit, which will exhibit a peak of respiration and ethylene production at the start of fruit ripening (Figure 4). Although very few ethylene release of tomato fruits was detected in DAA30, S10 and S12 fruits displayed higher average ethylene levels than those of CK and S6. Ethylene production in tomato fruits progressively increased with tomato ripening from DAA34 to DAA46. The fruits under blue light frequencies treatment maintained a higher ethylene production than CK, which exhibited the following trend: S10 > S12 > S8 > S6 > CK and the highest levels were found in S10.

### Supplemental Blue Light Frequencies Treatments Improve Fruit Quality

**FIGURE 5:**
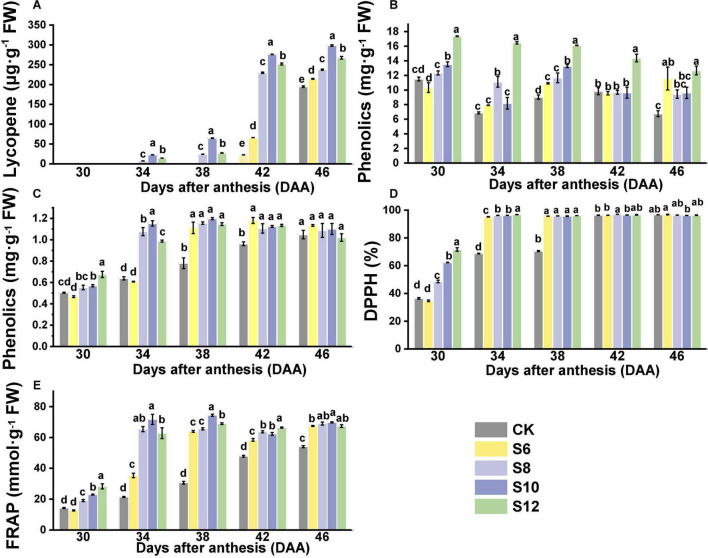
Effects of different supplemental blue light frequencies on the **(A)** lycopene content, **(B)** phenolics content, **(C)** flavonoids content, **(D)** 2,2-diphenyl-1-picrylhydrazyl (DPPH), and **(E)** ferric-reducing antioxidant power (FRAP) of tomato fruit. Values are means ± SD (*n* = 3). Different letters denote significant differences among treatments at each sampling day after anthesis separately (*p* ≤ 0.05).

Supplemental blue lighting had a positive effect on improving the contents of healthy compounds and antioxidant capacity (Figure 5). The lycopene content in tomato fruits showed an upward trend with fruit gradually developing and ripening (Figure 5A). At DAA30, the lycopene contents were virtually undetectable in any tomato fruits that still kept green. Lycopene contents in CK and S6 did not to be detected until DAA42 and displayed much lower lycopene contents than S8, S10, and S12 at DAA42 and DAA46. Lycopene contents of fruits under supplemental blue lighting were significantly higher than those of CK, which exhibited the trend as follows: S10 > S12 > S8 > S6 (Figure 5A). The values of DPPH and FRAP under blue light treatment were higher than those of the CK fruits with the fruits ripening, except for that no striking differences were found in DPPH between treated fruits and CK fruit at DAA42 and DAA46 (Figures 5B,C). Moreover, the higher contents of flavonoids and phenolics were found in the supplementary blue light compared with CK and the highest flavonoids contents were discovered in S12 (Figures 5D,E). From DAA30 to DAA38, there was a trend that antioxidant compound contents and capacity increased with the duration of blue light increasing among the blue light treatment, but no obvious differences were observed in the ripening stage of tomato fruit (except for flavonoids).

**FIGURE 6:**
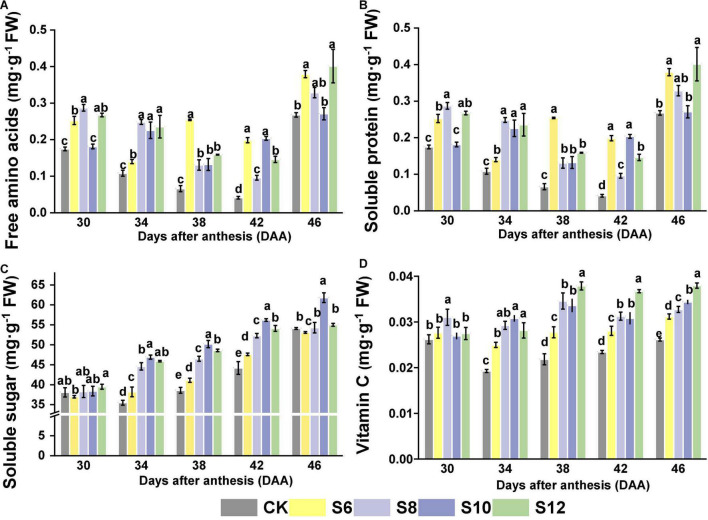
Effects of different supplemental blue light frequencies on the **(A)** free amino acids content, **(B)** soluble protein content, **(C)** soluble sugar content, and **(D)** vitamin C content of tomato fruit. Values are means ± SD (*n* = 3). Different letters denote significant differences among treatments at each sampling day after anthesis separately (*p* ≤ 0.05).

A positive tendency of nutritional and overall sensory profiles of tomato fruits was detected in supplemental blue light treatments. The content of free amino acids in tomato fruits had no significant difference from those of CK under blue light treatment at DAA30, but significantly higher than those of CK at DAA46, while no uniform performances were found in DAA34, DAA38, and DAA42 (Figure 6A). Supplemental blue lighting induced an increasing trend in soluble protein contents, except for those under S10 at DAA30 and DAA46, which exhibited no statistically significant difference compared with CK (Figure 6B). An increasing trend was observed in soluble sugar content under all the treatments with the fruit ripening. Supplemental blue light had a greater positive effect on the soluble sugar content compared with CK, a significant increase in the soluble sugar content was observed in all the blue light treatments, and S10 presented the greatest effect on the soluble sugar content during the tomato fruit ripening (Figure 6C). Vitamin C contents gradually increased with the increasing duration of blue light and the greatest values were under S12 in various stages of fruit development (Figure 6D).

## Discussion

### Different Frequencies of Supplemental Blue Light Accelerated Flowering and Fruit Ripening of Tomato Plants

Light plays a very vital role in tomato flowering, fruit developing, and ripening. Photoreceptors are involved in the control of flowering time, which are strictly associated with light fluence and photoperiod (Giliberto et al., 2005; Fantini et al., 2019). When plants exposure to blue light, the photoexcited photoreceptor CRY2 interacts with SPA1, which enhances CRY2-COP1 interaction to suppress COP1 activity and CO degradation and consequently mediated floral initiation in response to photoperiodic signals (Liu et al., 2011). In this study, supplementary blue light resulted in earlier flowering in tomato (Figure 1), which might be depend on the expression of flowering-associated genes (e.g., *FT*, *SOC1*, and *CO*) that mediated by cryptochromes (Giliberto et al., 2005; Fantini and Facella, 2020). Higher frequencies of supplemental blue light accelerated tomato flowering that might be related to the amount and timing of blue light they received.

Color is the predominant visual characteristic for tomato fruit ripening. Colorimetric parameters (value of L*, a*, b*, hue, and chroma) displayed a positive correlation with the ripening stages of tomato fruit. The a* and b* value increased during tomato ripening as a consequence of lycopene biosynthesis and chlorophyll depletion, representing the color change from green to red. From DAA34 to DAA42, tomato fruits under S6, S8, S10, and S12 maintained higher a*/b* (yellowness) and lower value of hue and chroma than CK, particularly in S10 (Figure 3), indicating that different frequencies of supplemental blue light markedly accelerated tomato fruit ripening initiation and progression.

Tomato fruit is a typical climacteric fruit, which displays an obvious characteristic that ethylene production will dramatically increase at the onset of fruit ripening (Alexander and Grierson, 2002). Ethylene emission and the alterations in transcriptional profile of ethylene biosynthetic genes in tomato fruits were tightly regulated by light (Wilson et al., 2014; Harkey et al., 2019; Seo and Yoon, 2019). Supplementary red light promoted ethylene biosynthesis, leading to the accumulation of phytoene and the earlier ripening in tomato fruit (Zhang et al., 2020). As reported elsewhere, blue light strongly upregulated the expression in *PpACO1*, *PpACS3*, *PpEIN2*, *PpEIL1*, *PpEIL2*, and *PpERF2*, which function in ethylene signaling and biosynthetic pathway, promoted ethylene production and fruit softening in peaches (Gong et al., 2015). In this study, we observed ethylene burst under different supplemental light treatments from DAA34, which exhibited the following trend under supplemental light treatments: S10 > S12 > S8 > S6 > CK (Figure 4). Additionally, a rapid change in fruit color was found in S10, S12, and S8, especially in S10, suggesting that higher frequencies of supplemental blue light accelerated tomato fruit ripening were well associated with ethylene production.

### Different Frequencies of Supplemental Blue Light Improved Antioxidant Compound Contents and Capacity of Tomato Fruits

Lycopene, as the predominant carotenoid in the tomato fruit, is responsible for the red color of fruit. There is a significant increase in lycopene contents when fruits turn red from breaker stage. In this study, lycopene content in fruits under S8, S10, and S12 treatments increased rapidly from DAA34 to DAA46 (Figure 5A). Moreover, the fruits under different frequencies of supplemental blue light displayed higher lycopene contents than those in CK and S10 maintained the highest contents (Figure 5A). A previous study indicated that blue LEDs promoted tomato ripening along with induction of the synthesis of β-carotene, lutein, α-tocopherol, and γ-tocopherol (Gong et al., 2015). Also, supplemental blue lighting significantly increased the expression of key genes (*PSY1*, *CRTISO*, and *VDE*) that involved in lycopene biosynthesis (Wang et al., 2021). In addition, the phytoene, lycopene, and ß-carotene content in tomato fruits were higher in OE-CRY1a lines than those in WT and *cry1a* plants, indicating that *CRY1a*-mediated blue light signal is critical for regulating fruit pigmentation (Liu et al., 2018). A previous study indicated that the photosynthetic rate for various crops responded differently to pulsed lighting and highly depended on the frequency and duty ratio of pulsed light (Son et al., 2016).

The carotenoids contents of lettuce were sharply enhanced by pulsed lighting due to pulsed light and had a remarkable effect on increasing photosynthetic rate in lettuce (Miliauskienė et al., 2021). Thus, different frequencies of supplemental blue light could promote the early color change and maturation of tomato fruits; this could be attributed to blue light regulating the transcriptional levels of light transcription factors such as *SlPIFs* and *SlHY5* gene and mature promoter gene *SlRIN*, thus affecting the process of fruit lycopene metabolism (Xie et al., 2019; Dong et al., 2021). Appropriate pulsed lighting might be beneficial for light capture and light energy conversion in the light reactions and carbon capture and conversion in the dark reaction, which together are important for carotenoid production in the tomato fruits.

Phenolics and flavonoids are health-protecting components in the human diet as a result of their capacity to activate endogenous antioxidant defense systems. More specifically, blue light was effective in stimulating simple phenols and flavonoids contents in many horticultural crops (Kim et al., 2014; Nam et al., 2018; Matysiak and Kowalski, 2019; Xie et al., 2020). The activity of key enzymes in shikimate and phenylpropanoid pathways, such as phenylalanine ammonia lyase (PAL), chalcone synthase (CHS), and chalcone isomerase (CHI) or in flavonoid synthesis pathways, such as flavonol synthase (FLS), could be significantly enhanced by blue light (Maroga et al., 2019; Wilawan et al., 2019). Phenolic compounds of buckwheat sprouts showed higher accumulation under blue light than other lights, with higher expression of the *FtPAL*, *FtF3’H*, *FtC4H*, *FtCHI*, *FtFLS-2*, and *FtANS*, which were responsible for flavonoids accumulation increased dramatically (Thwe et al., 2014). Similarly, compared with white and red LED irradiation, blue LED (470 nm, 100 μmol m^–2^ s^–1^) resulted in a significant increase in the contents of C-glycosylflavone (saponarin, isoorientin, and isoschaftoside) and saponarin, which constituted the major portion of flavonoids detected in barley and wheat sprouts (Muthusamy et al., 2020). Cryptochromes (CRYs) are blue light photoreceptors found in plants. CRY2 overexpression increased flavonoid and lycopene levels in ripe tomato fruits (Giliberto et al., 2005). Compared to continuous lighting, pulsed light increased polyphenols contents and the antioxidant capacity of lettuce (Miliauskienė et al., 2021) and wheat plants (Dong et al., 2015), which could be due to the stimulating effect of pulsed light on gene expression and activity rates of the key enzymes related to phenylpropanoid biosynthesis. In this study, the phenolics and flavonoids content under supplementary blue light treatments were significantly higher than CK (Figures 5B,C) and higher levels of phenolics and flavonoids were observed in higher frequencies of supplemental blue light (Figures 5B,C). These results might be due to higher frequencies of supplemental blue light upregulated the expression of genes expression, which regulate flavonoid synthesis *via* light receptors. Thus, changes in phenolic and flavonoid compounds in tomato fruits depended upon the pulse frequencies of blue light.

2,2-diphenyl-1-picrylhydrazyl and FRAP are generally used to evaluate the antioxidative capacities of vegetables and compounds such as Vc, carotenoids, phenolics, and flavonoids significantly contribute to the total antioxidant capacity. The DPPH and FRAP in two pak-choi cultivars were affected by blue light illumination (400 and 430 nm) (Mao et al., 2021). In this study, higher values of DPPH and FRAP were found in fruits under blue lighting treatment, which might be related to the increase of phenolics and flavonoids contents in fruits under supplemental blue lights. Compared with CK, DPPH and FRAP were pronouncedly increased with the blue light frequencies increasing (Figures 5C,D), which was possibly related to the longer duration of pulsed blue light.

### Different Frequencies of Supplemental Blue Light Improved the Nutritional Compounds of Tomato Fruits

Amino acids are one of the essential components for plants to complete their life cycle, in which metabolism is highly related to light environment. Blue light enhanced the formation of phenylalanine *via* increasing PAL activity in okra treated by blue light, whereas it reduced the tryptophan content compared with white light (Wilawan et al., 2019). Higher concentration of proline accumulated in tomato and wheat treated by blue light (Hee-Sun Kook, 2013; Monostori et al., 2018). In this study, free amino acid contents in tomato fruits at red ripening stage (DAA42 and DAA46) were higher in all the blue lighting treatments than CK, which nearly exhibited an increasing trend with higher blue frequencies, except for DAA38 (Figure 6A). Meanwhile, beneficial effects of blue light on soluble protein contents were found, although no uniform pattern among treatments in different frequencies (Figure 6B). Blue light was favorable for enhancing photosynthesis, which benefited for providing a nitrogen source and stimulating the metabolism of nitrate (Bian et al., 2015; Wang et al., 2015). Afterward, amino acids can be transformed from nitrate through enzyme catalysis, which provides substrates for the synthesis of soluble proteins (Wang et al., 2018).

Soluble sugar contents in tomato fruit are closely linked to the sweetness and crispness of fruits, which could be derived from carbohydrate accumulation induced by light environment. Soluble sugar accumulation under blue light might be due to photosynthetic carbon partitioning as well as related to stomatal aperture and amount of stomata, which displayed a high sensitivity to blue light (Fantini et al., 2019). A previous study showed that tomato seedlings under continuous monochromatic blue light displayed the lowest contents of starch and soluble sugars, compared to the other treatments due to leaf injuries caused by continuous light (Pham et al., 2019). Intermittent illumination with 1, 2, 4, 6, and 8 L/D cycles, respectively, over the period showed that higher levels of soluble sugar, especially fructose and starch, were observed in lettuce exposed to L/D (2) and L/D (3) compared with any other treatment, *via* increasing activities of sucrose synthase (SS), acid invertase (AI), and neutral invertase (NI) involved in sucrose metabolism (Chen and Yang, 2018). Soluble sugar content increased drastically under blue light with development of fruits (from DAA34 to 46), where these displayed an increasing trend with the frequencies increasing and reached the maximum at S10 (Figure 6C). It implied appropriate intermittent irradiation that might influence sugar compositions *via* stimulating the activities of sucrose metabolism enzymes. Thus, S10 might induce a stronger metabolism of the imported sucrose, thus allowing more sucrose to be imported into the fruit that might attribute to photosynthesis enhancement induced by blue light (Koch, 2004).

Vitamin C mainly acts as an antioxidant, participates in various biological and metabolic processes of almost all the living organisms, and plays a critical role against diverse diseases. The accumulation of vitamin C was more pronouncedly triggered by blue light supplementation (430 and 465 nm) in Chinese kale and pak-choi baby leaves (Li et al., 2020). Vitamin C biosynthesis-related genes, regeneration genes, and two glutathione (GSH)-producing genes were upregulated by blue LED in three citrus varieties (Zhang et al., 2015). Furthermore, continuous irradiation with blue LED light was more effective than pulsed irradiation for increasing the vitamin C content in the juice sacs of three citrus varieties (Zhang et al., 2015). Additionally, pulsed light can provide higher photosystem II quanta efficiency and plant productivity compared to continuous light, leading to an enhancement in the synthesis and accumulation of hexose and D-glucose, which act as the precursors of vitamin C (Ntagkas et al., 2019). In this study, the vitamin C content of tomato fruits revealed a similar trend with soluble sugar levels, where its accumulation was more pronouncedly triggered by extended illumination time (Figure 6D). Our result indicated that blue LEDs provide a good strategy to improve the contents of vitamin C in tomato fruits, which displayed an increasing trend with the frequencies increasing, although the highest content was observed in S12.

## Conclusion

Different frequencies of supplemental blue light could accelerate flowering of tomato plants, dramatically increase the ethylene production, promoting fruit ripening about 3–4 days ahead, which significantly facilitated the processes of color change and maturity in tomato fruits. Higher nutritional and overall sensory profiles of tomato fruits were detected in supplemental blue light treatments, with a significant increase in the contents of lycopene, total phenolic compounds, total flavonoids, vitamin C, and soluble sugar, as well as the overall antioxidant activity of tomato fruits.

Different frequencies of supplemental blue light could promote the early color change and maturation of tomato fruits and significantly reinforced the antioxidant levels and nutritional qualities of tomato fruits, especially lycopene. Among them, S10 (30 min blue and 8 min pause, 100 μmol m^–2^ s^–1^ 430 nm blue LED light supplemented from 6:00 to 18:00) treatment was more efficient for tomato fruits production in the greenhouse. In addition, understanding the effects of different supplemental blue light frequencies on tomato needs to more comprehensive and strict exploration.

## Data Availability Statement

The original contributions presented in the study are included in the article/supplementary material, further inquiries can be directed to the corresponding author.

## Author Contributions

RH performed the experiments, analyzed the results, and wrote the manuscript. JW, JZ, MG, and YL performed the experiments and statistical analysis. HL conceived and designed the experiments, analyzed the results, contributed to manuscript revision, and read and approved the submitted version of the manuscript. All authors have read and approved the manuscript for publication.

## Conflict of Interest

The authors declare that the research was conducted in the absence of any commercial or financial relationships that could be construed as a potential conflict of interest.

## Publisher’s Note

All claims expressed in this article are solely those of the authors and do not necessarily represent those of their affiliated organizations, or those of the publisher, the editors and the reviewers. Any product that may be evaluated in this article, or claim that may be made by its manufacturer, is not guaranteed or endorsed by the publisher.
